# Supplementary figures and images for: 2-Bromopalmitate decreases spinal inflammation and attenuates oxaliplatin-induced neuropathic pain via reducing Drp1-mediated mitochondrial dysfunction
Source: PLoS One. 2022 Oct 31;17(10):e0275428. doi: 10.1371/journal.pone.0275428 (PMC9621438; doi:10.1371/journal.pone.0275428)

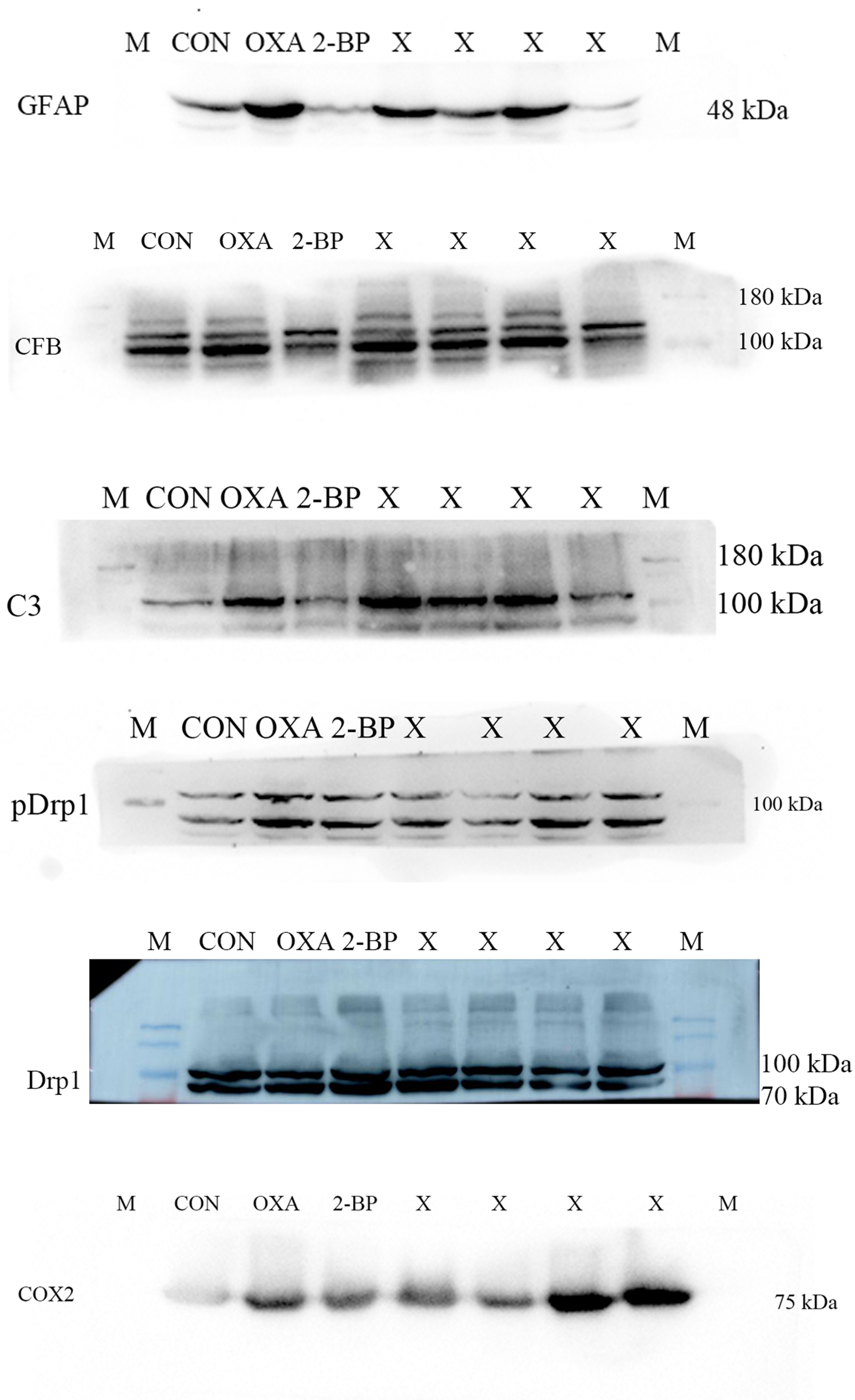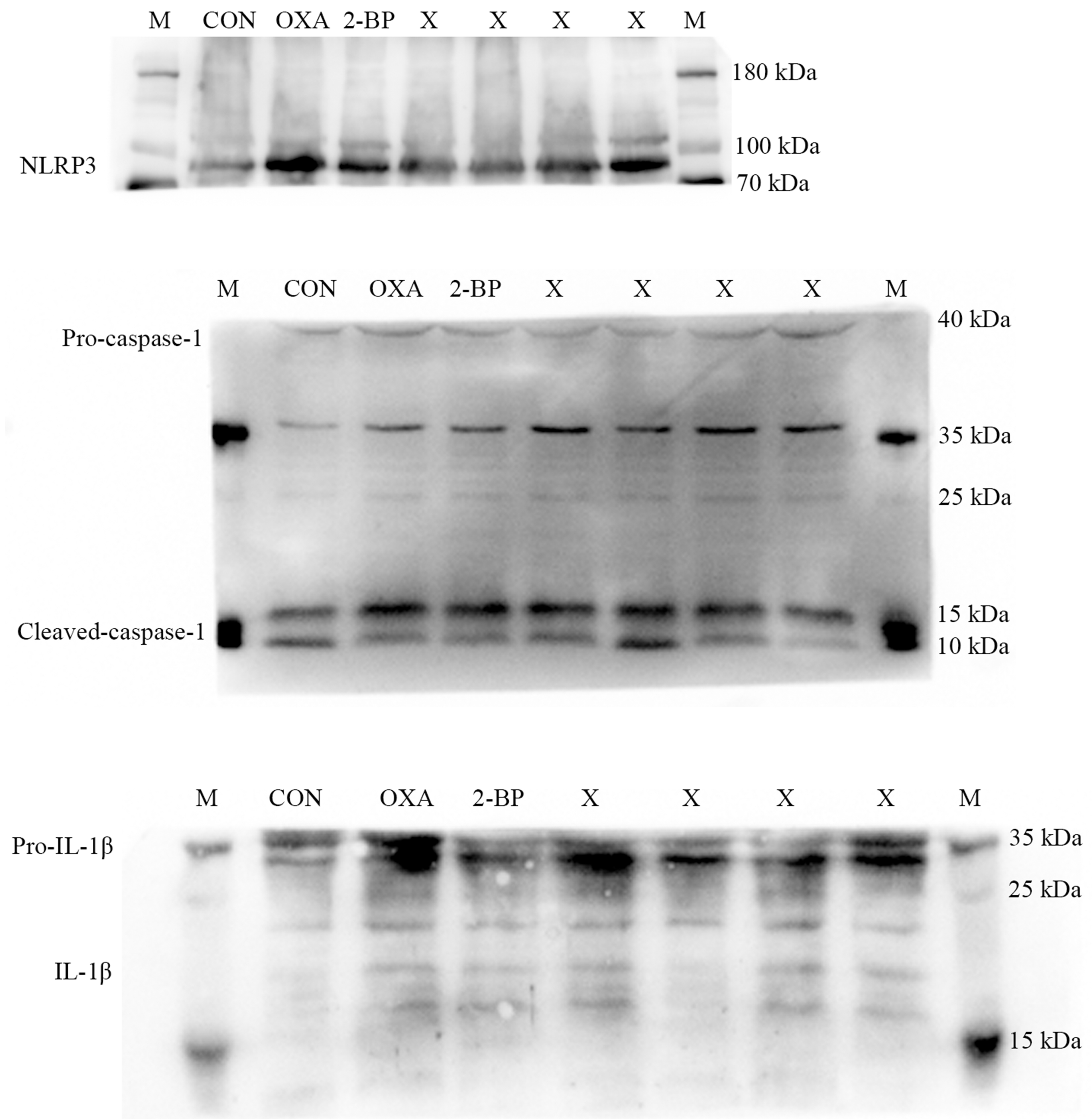

Supplement: S1 File — (PDF) [file pone.0275428.s002.pdf]
