# Supplementary material for: 2-Bromopalmitate decreases spinal inflammation and attenuates oxaliplatin-induced neuropathic pain via reducing Drp1-mediated mitochondrial dysfunction
Source: PLoS One. 2022 Oct 31;17(10):e0275428. doi: 10.1371/journal.pone.0275428 (PMC9621438; doi:10.1371/journal.pone.0275428)
Supplement: Attachment — Submitted filename: Response to Reviewers.docx [file pone.0275428.s003.docx]

Dear Editor Rosanna Di Paola

Thank you for seeding the Reviewers’ and the Editors’ comments of our paper entitled “2-Bromopalmitate decreases spinal inflammation and attenuates oxaliplatin-induced neuropathic pain via reducing Drp1-mediated mitochondrial dysfunction”. The insightful comments and constructive suggestions are extremely useful in significantly improving our study, allowing us to prepare a much stronger manuscript. We are now submitting a revised manuscript. A detailed point-by-point response to Reviewers’ and Editors’ comments were presented below. The bold text portions are the comments, and our response follows each comment.

**Journal Requirements:**

**When submitting your revision, we need you to address these additional requirements.**

**1. Please ensure that your manuscript meets PLOS ONE's style requirements, including those for file naming. The PLOS ONE style templates can be found at**

**https://journals.plos.org/plosone/s/file?id=wjVg/****PLOSOne_formatting_sample_main_body.pdf and**

**https://journals.plos.org/plosone/s/file?id=ba62/PLOSOne_formatting_sample_title_authors_affiliations.pdf**

Response: We have modified the article format according to the PLOSOne formatting sample title authors affiliations and PLOSOne formatting sample main body templates in the revised manuscript.

**2. As part of your revision, please complete and submit a copy of the Full ARRIVE 2.0 Guidelines checklist, a document that aims to improve experimental reporting and reproducibility of animal studies for purposes of post-publication data analysis and reproducibility:**

**https://arriveguidelines.org/sites/arrive/files/Author%20Checklist%20-%20Full.pdf (PDF). Please include your completed checklist as a Supporting Information file. Note that if your paper is accepted for publication, this checklist will be published as part of your article.**

Response: We completed the Checklist and uploaded as a Supporting Information file.

**3. We note that the grant information you provided in the ‘Funding Information’ and ‘****Financial Disclosure’ sections do not match.**

Response: We have checked and corrected the Funding Information in the revised manuscript.

**4. Thank you for stating the following in the Funding Section of your manuscript:**

**“This study was supported by the grants from the National Natural Science Foundation of China (Nos. 81971066, 81901149 and 3210070630), Research Project of Hubei Provincial Department of Education (Nos. Q20192807, B2019167), Hubei University of Science and Technology Program (Nos. 2020TD02, BK20116).”**

**We note that you have provided additional information within the Acknowledgements Section that is not currently declared in your Funding Statement. Please note that funding information should not appear in the Acknowledgments section or other areas of your manuscript. We will only publish funding information present in the Funding Statement section of the online submission form.**

**Please remove any funding-related text from the manuscript and let us know how you would like to update your Funding Statement. Currently, your Funding Statement reads as follows:**

**“The funders had no role in study design, data collection and analysis, decision to publish, or preparation of the manuscript.”**

**Please include your amended statements within your cover letter; we will change the online submission form on your behalf.**

Response: We have modified the Funding Information according to the PLOSOne formatting sample main body templates and deleted the Acknowledgements in the revised manuscript.

We also have added the “The funders had no role in study design, data collection and analysis, decision to publish, or preparation of the manuscript” to the Funding part in the revised manuscript.

The amended statements were added in the cover letter.

**5. We note that you have indicated that data from this study are available upon request. PLOS only allows data to be available upon request if there are legal or ethical restrictions on sharing data publicly. For more information on unacceptable data access restrictions, please see http://journals.plos.org/plosone/s/data-availability#loc-unacceptable-data-access-restrictions.**

**In your revised cover letter, please address the following prompts:**

**a) If there are ethical or legal restrictions on sharing a de-identified data set, please explain them in detail (e.g., data contain potentially sensitive information, data are owned by a third-party organization, etc.) and who has imposed them (e.g., an ethics committee). Please also provide contact information for a data access committee, ethics committee, or other institutional body to which data requests may be sent.**

**b) If there are no restrictions, please upload the minimal anonymized data set necessary to replicate your study findings as either Supporting Information files or to a stable, public repository and provide us with the relevant URLs, DOIs, or accession numbers. For a list of acceptable repositories, please see http://journals.plos.org/plosone/s/data-availability#loc-recommended-repositories.**

**We will update your Data Availability statement on your behalf to reflect the information you provide.**

Response: The Data Availability Statement in the revised manuscript was amended as follows: All relevant data are within the paper.

**6. PLOS requires an ORCID iD for the corresponding author in Editorial Manager on papers submitted after December 6th, 2016. Please ensure that you have an ORCID iD and that it is** **validated in Editorial Manager. To do this, go to ‘Update my Information’ (in the upper left-hand corner of the main menu), and click on the Fetch/Validate link next to the ORCID field. This will take you to the ORCID site and allow you to create a new iD or authenticate a pre-existing iD in Editorial Manager. Please see the following video for instructions on linking an ORCID iD to your Editorial Manager account: https://www.youtube.com/watch?v=_xcclfuvtxQ.**

Response: The corresponding author has a validated ORCID iD.

**7. PLOS ONE now requires that authors** **provide the original uncropped and unadjusted images underlying all blot or gel results reported in a submission’s figures or Supporting Information files. This policy and the journal’s other requirements for blot/gel reporting and figure preparation are described in detail at https://journals.plos.org/plosone/s/figures#loc-blot-and-gel-reporting-requirements and https://journals.plos.org/plosone/s/figures#loc-preparing-figures-from-image-files. When you submit your revised manuscript, please ensure that your figures adhere fully to these guidelines and provide the original underlying images for all blot or gel data reported in your submission. See the following link for instructions on providing the original image data: https://journals.plos.org/plosone/s/figures#loc-original-images-for-blots-and-gel**

Response: The original uncropped and unadjusted images underlying all blot or gel results were uploaded.

**8. We note that you have included the phrase “data not shown” in your manuscript. Unfortunately, this does not meet our data sharing requirements. PLOS does not permit references to inaccessible data. We require that authors provide all relevant data within the paper, Supporting Information files, or in an acceptable, public repository. Please add a citation to support this phrase or upload the data that corresponds with these findings to a stable repository (such as Figshare or Dryad) and provide and URLs, DOIs, or accession numbers that may be used to access these data. Or, if the data are not a core part of the research being presented in your study, we ask that you remove** **the phrase that refers to these data.**

Response: The phrase “data not shown” was deleted in the revised manuscript.

**9. Your ethics statement should only appear in the Methods section of your manuscript. If your** **ethics statement is written in any section besides the Methods, please move it to the Methods section and delete it from any other section. Please ensure that your ethics statement is included in your manuscript, as the ethics statement entered into the online submission form will not be published alongside your manuscript.**

Response: The ethics statement was added in the Methods section in the revised manuscript.

**10. Please review your reference list to ensure that it is complete and correct. If you have cited papers that have been retracted, please include the rationale for doing so in the manuscript text, or remove these references and replace them with relevant current references. Any changes to the reference list should be mentioned in the rebuttal letter that accompanies your revised manuscript. If you need to cite a retracted article, indicate the article’s retracted status in the References list and also include a citation and full reference for the retraction notice.**

Response: We checked the reference and corrected the wrongly cited literature and cited some latest literatures.

**Reviewers' comments:**

**General comments:**

**The paper by Zhi-Bin Dong and colleagues add another important element regarding the toxicity of platinum derivate Oxaliplatin and on the use of 2-Bromopalmitate to counteract these effects. This manuscript represents a good starting point as reference data for future studies on this topic. Despite the present study has a good experimental idea, it needs revisions both for contents and structural organization in order to improve the quality of the manuscript. Some acronyms are missing, written in full, eg: DCFH-DA and PWT, please add them in the text.**

Response: Both the contents and structural organization has been modified and the full written of the abbreviations has been added in the revised manuscript. In addition, the keywords have been adjusted.

**Keywords:**

**It would be advisable to change the keywords; some of these are also present in the paper's title.**

Response: The keywords in the revised manuscript were “chemotherapy pain, oxaliplatin, 2-bromopalmitate, NLRP3 inflammasome, dynamin-related protein 1 (Drp1), manganese superoxide dismutase (Mn-SOD), cyclooxygenase-2 (COX-2)”

**Background:**

**It would be appropriate to include more information about oxaliplatin toxicity, so I suggest that the authors include the following reference on zebrafish model in which many aspects related to oxaliplatin toxicity from both morphological and molecular points of view are emphasized. DOI: 10.3390/toxics10020081.**

Response: The oxaliplatin toxicity on the changes in the tissues was added in the Introduction section of the revised manuscript as follows: “As an anti-cancer agent, OXA induced histopathological changes in the heart, liver, intestines and muscle in a dose dependent manner”.

**Materials and Methods;**

**Materials and Methods section needs revision:**

**It would be opportune in indicate the full addresses of the manufacturers from whom the materials used for conducting the experiments are procured, please standardize the whole section accordingly.**

Response: The full address of the manufacturers and catalogue number of the antibodies and kits were added in the Antibodies and reagents section.

**It would be desirable to improve the quality of the description of the methods used as well, I suggest taking a cue for the exposition of the Western Blot procedure from the following manuscript and insert the appropriate citation : DOI: 10.3390/life12010128**

Response: The details of Western Blot procedure were added according to the reference (DOI: 10.3390/life12010128) in the Western blot analysis section.

**The authors need to implement the quality of even the purely technical sections, for SOD measurement take a cue and cite: DOI: 10.3390/toxins13100710**

**Same for ROS assessment section, take a cue from the text and cite it: DOI: 10.3390/toxics9120344**

Response：The details of SOD and ROS measurements were added and the references were cited in the Assessment sections of revised manuscript.

**The authors stated: “After TNF-α inducement and 2-BP treatment, C6 cells were loaded with MitoTracker Red CMXRos or DCFH-DA and incubated at 37 ℃ for 30 min”.It’s not clear what the author used for this assay, was MitoTracker Red CMXRos or DCFH-DA?,please revise.**

Response: The mitochondrial membrane potential measurement and ROS measurement have been described respectively as two separated sections and the details of the processes were added in the revised manuscript.

**Figures: I suggest the authors to arrange the legend figures ,sometimes there are letters corresponding to a photo in brackets,sometimes not,it becomes very difficult to follow what the authors are trying to describe.**

Response: The letters represented the figures of photo have been added in the descriptions.
